# Associations of serum gamma-linolenic acid levels with erythema severity and anxiety/depression status in patients with rosacea^☆^

**DOI:** 10.1016/j.abd.2023.01.008

**Published:** 2023-12-06

**Authors:** Jin-Yi Tang, Mei-Ling Chen, Mei Wan, Jin-Yu Wei, Tian Qian, Yu-Kun Fan, Zhi Yang, Jian Fu, Jian Li

**Affiliations:** a Office of Scientific Research Administration, Division of Medical Affairs, Southwest Hospital, Third Military Medical University, Chongqing, China; b Nuclear Medicine Department, Southwest Hospital, Third Military Medical University, Chongqing, China; c Dermatology Department, Southwest Hospital, Third Military Medical University, Chongqing, China; d Dermatology Department, The 920th Hospital of Joint Logistics Support Force of PLA, Kunming, China; e Dermatology Department, The Third Affiliated Hospital of Chongqing Medical University, Chongqing, China; f Urology, Southwest Hospital, Third Military Medical University, Chongqing, China

**Keywords:** Gamma-linolenic acid, Mental disorders, Rosacea, Severity of illness index

## Abstract

**Background:**

The development of rosacea is suggested to be closely associated with lipid metabolism, inflammation, and anxiety/depression. Gamma linolenic acid (GLA) is a key factor participating in lipid metabolism, which is also confirmed to regulate the inflammatory response. However, the associations of serum GLA levels with rosacea severity and psychological status still remain unclear.

**Objective and limitations of the study:**

The present study aimed to investigate the associations of gamma linolenic acid (GLA), a key factor participating in lipid metabolism and the inflammatory response, with rosacea severity and psychological status. The present study still had some limitations. First, this study is a cross-sectional study and does not provide longitudinal evidence about the relationship between GLA and rosacea; Second, the cohort in this study is also relatively small, and a larger cohort is needed in further investigation to reveal the potential role of lipid metabolism in the pathogenesis of rosacea.

**Methods:**

A total of 62 rosacea patients were consecutively recruited. Patient’s Self-Assessment (PSA) scale and Clinician Erythema Assessment (CEA) as well as 7-item Generalized Anxiety Disorder (GAD-7) and 9-item Patient Health Questionnaire (PHQ-9) were conducted to evaluate the degree of erythema severity and anxiety/depression, respectively. Serum GLA levels were determined by gas chromatography mass.

**Results:**

Lower levels of serum GLA in rosacea patients were observed (p < 0.001), and subgroup analysis revealed that patients with higher-level GLA had lower scores of PSA, CEA, GAD-7 and PHQ-9. Moreover, Spearman correlation analysis uncovered that serum GLA levels were negatively associated with PSA, CEA, GAD-7 as well and PHQ-9 scores, respectively. Linear regression model found that serum GLA levels at baseline were a predictive factor for prognosis of clinical outcomes after 1-month conventional treatment.

**Conclusion:**

The present study indicates that lower levels of serum GLA in rosacea patients are negatively associated with the degree of erythema and anxiety/depression status.

## Introduction

Rosacea is a prevalent chronic dermatosis among adults, especially females.^1^ Rosacea is currently segmented into four subtypes, including erythaotelangiectactic rosacea (ETR), papulopustular rosacea (PPR), phymatous rosacea (PHY), and ocular rosacea (OR). The most prevalent subtype is ETR,^2^ in which, the most common symptoms are persistent erythema and flushing, bringing a considerably negative impact on rosacea patients. In recent years, rosacea has begun to occur in the younger, which might cause more detrimental consequences, such as low working enthusiasm and anxiety/depression.^3^ However, except for topical brimonidine, there are no other approved treatments due to the poor understanding of the mechanism of rosacea.

Although the mechanism is not fully understood, current advances suggest that rosacea is a complicated disease influenced by multifaced risk factors, including immune response, anxiety/depression, impaired skin barrier, and abnormally local or systematical lipid metabolism.^4^, ^5^, ^6^, ^7^ Specifically, the compromised epidermal permeability barrier induces the production and release of inflammatory factors and infiltration of immune cells, consequently causing skin dryness, stinging, and burning in rosacea patients.^8^ In addition, a previous study found that the composition of sebaceous lipids alters in rosacea patients in comparison with normal controls,^7^ suggesting the potential role of lipid metabolism in the pathogenesis of rosacea. Rosacea is also proposed as a chronic inflammatory skin disorder and activated inflammation can explain many of its signs and symptoms.^6^ The previous study found that anxiety/depression status plays a substantial role in the development of rosacea and regulating anxiety/depression status can facilitate the therapeutic efficacy of the current drugs.^3^ Thus, targeting these multiple pathways might hold promise for alleviating rosacea.

Gamma linolenic acid (GLA) is an endogenic n-6 polyunsaturated fatty acid (n-6 PUFA) that plays a crucial role in reducing lipid levels.^9^ A recent randomized clinical trial indicated that GLA as a supplement can improve clinical outcomes and reduce the lipid levels in patients with rosacea,^10^ highlighting that GLA might be involved in the pathogenesis of rosacea. To the best of our knowledge, few studies have been done to figure out the relationship between the development of rosacea and endogenic GLA levels. Therefore, in this study, the authors aimed to investigate the alternation of serum GLA levels and explore the association of serum GLA levels with erythema degree and anxiety status in patients with ETR.

## Subjects and methods

A total of 62 rosacea patients (ETR subtype) and 14 age-matched Normal Controls (NCs) were consecutively recruited from the southwest hospital. The authors collected their blood samples and examined blood GLA levels. In addition, the authors also assessed the erythema degree and anxiety status and their correlations with blood GLA levels, respectively. The detailed information was presented in supplementary data.

## Results

### Demographic characters

Table 1 shows the demographic characteristics of rosacea patients and NCs. As shown, there were no significant differences in age, gender, BMI, the prevalence of hypertension and diabetes as well as hypercholesterolemia between rosacea patients and NCs (Table 1, p > 0.05). In addition, serum GLA levels in rosacea patients at baseline were lower than those in NCs (24.66 ± 8.20 vs. 15.81 ± 10.58 moL/L, p = 0.004).

**Table 1:** Demographic characteristics.

Variable	Normal controls	Rosacea	Statistics	P-value
No. of patients	14	62		
Age (years)	34.64 ± 10.38	34.19 ± 10.92	t = 0.140	0.889
Male/female	9(5)	20 (42)	χ^2^=0.62	0.804
BMI (kg/m^2^)	23.21 ± 4.19	22.98 ± 5.34	t = 0.337	0.737
Education level (years)	12.34 ± 3.25	11.84 ± 2.97	t = 1.130	0.260
Hypertension (%)	4 (6.5)	10 (16.1)	χ^2^=1.177	0.278
Diabetes (%)	1 (7.1)	6 (9.7)	χ^2^=0.088	0.767
GLA levels (mol/L)	24.66 ± 8.20	15.81 ± 10.58	t = 2.933	0.004
CEA scores	NA	2.58 ± 1.00		
PSA scores	NA	2.90 ± 0.74		
GAD-7 scores	NA	6.56 ± 4.98		
PHQ-9 scores	NA	5.10 ± 4.33		

Note: NA denotes not applicable.

### Comparisons of CEA and PSA scores between the low-level GLA group and the high-level GLA group among rosacea patients

**Figure 1:**
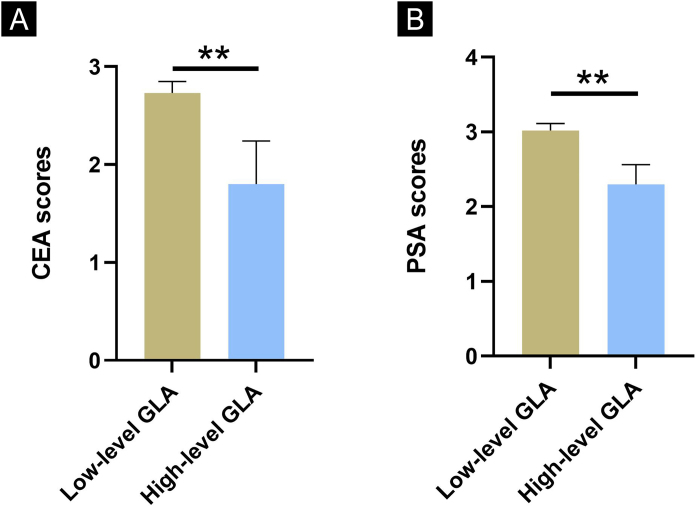
Comparisons of CEA and PSA scores between the low-level GLA group and the high-level GLA group. (A) Comparison of CEA scores. (B) Comparison of PSA scores. ** Indicates p < 0.01; n = 53 for the low group; n = 9 for the high-level group. The error bars present the SD.

The authors used the median value of NC samples as the cutoff value to distinguish the high-level GLA group and low-level GLA group (26.32 moL/L). Subsequently, rosacea patients were divided into the low-level GLA group and the high-level GLA group according to the cutoff value. Next, the authors compared the CEA and PSA scores between the low-level GLA group and the high-level GLA group among rosacea patients. It was interesting to find out that rosacea patients with lower-level GLA had higher CEA and PSA scores than those in patients with higher-level GLA (Fig. 1, p < 0.01 for all).

### Correlations of GLA levels with PSA and CEA scores in patients with rosacea

**Figure 2:**
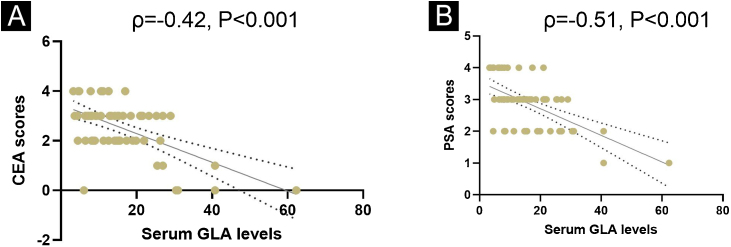
Correlations of GLA levels with PSA and CEA scores in patients with rosacea. (A) Correlation of serum levels with CEA scores. (B) Correlation of serum levels with PSA scores (n = 62 for each analysis).

ETR is a prevalent subtype of rosacea, whose most common symptom is erythema. PSA and CEA scores are usually applied to reflect the severity of erythema. To further uncover the association of GLA levels with the disease severity of rosacea, the authors conducted the Spearman correlation analysis. As illustrated in Fig. 2, serum GLA levels are strikingly associated with PSA scores as well as CEA scores (Fig. 2).

### Predictive value of GLA levels for CEA and PSA scores after 1-month treatment

To explore the predictive effects of GLA level at baseline for CEA and PSA scores after 1-month conventional treatment, the authors further conducted the linear regression analysis with CEA or PSA scores as the independent variable, respectively. The contributing factors include age, gender, hyperlipidemia, hypertension DM, CEA scores at baseline, and PSA scores at baseline. It is apparent that GLA levels at baseline were a significant predictive factor for CEA and PSA scores after 1-month follow-up, respectively (Table 2, Table 3).

**Table 2:** The predictive value of GLA levels for CEA scores after a 1-month of treatment.

Variables	Unstandardized coefficients		Standardized Coefficients	P value
	B	SD	Beta	
Constant	1.131	0.720		0.122
GLA levels	−0.059	0.014	−0.457	0.000
Age	0.016	0.018	0.126	0.375
Sex	0.275	0.300	0.095	0.362
Diabetes	0.386	0.514	0.085	0.456
hypertension	−1.302	0.472	−0.355	0.008
CEA at baseline	0.423	0.133	0.332	0.003

Note: SD denotes Standard deviation.

**Table 3:** The predictive value of GLA levels for PSA scores after a 1-month of treatment.

Variables	Unstandardized coefficients		Standardized Coefficients	P value
	B	SD	Beta	
Constant	0.066	.920		0.122
GLA levels	−.051	.014	−.448	0.001
Age	0.059	0.017	0.533	0.001
Sex	−0.415	0.030	−0.162	0.171
Diabetes	−0.844	0.537	−0.208	0.122
hypertension	−0.604	0.462	−0.185	0.197
PSA at baseline	0.158	0.206	0.097	0.046

Note: SD denotes Standard deviation.

### Correlations of GLA levels with GAD-7 and PHQ-9 scores in patients with rosacea

**Figure 3:**
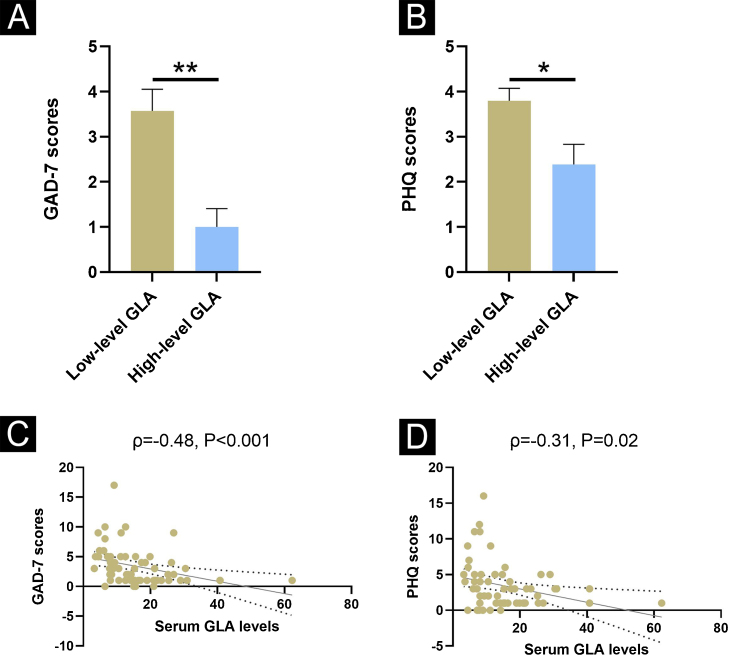
Correlations of GLA levels with PSA and CEA scores in patients with rosacea. (A) Comparison of GAD-7 scores. (B) Comparison of PHQ-9 scores. (C) Correlation of serum levels with GAD-7 scores. (D) Correlation of serum levels with PHQ-9 scores. * Indicates p < 0.05; ** Indicates p < 0.01; n = 53 for the low-group; n = 9 for the high-level group; n = 62 for the correlation analysis. The error bars present the SD.

As anxiety/depression has a direct impact on the development of rosacea, the authors further investigated the relationship between serum GLA levels and GAD-7 as well as PHQ-9 scores. Initially, the authors compared GAD-7 and PHQ-9 scores between the low-level GLA group and the high-level GLA group among rosacea patients. The low-level GLA group displayed higher GAD-7 and PHQ-9 scores than the high-level GLA group (Fig. 3A‒B, p < 0.05). In addition, Spearman correlation analysis was performed to figure out the association of GLA levels with GAD-7 and PHQ-9 scores. Interestingly, both GAD-7 and PHQ-9 scores were negatively correlated with GLA levels among patients with rosacea (Fig. 3C‒D).

## Discussion

In the present study, the authors aimed to investigate the association of serum GLA levels with erythema and anxiety/depression status. The authors found that rosacea patients had a higher level of GLA than NCs, and CEA and PSA scores as well as GAD-7 and PHQ-9 scores level in the low-level GLA ones were remarkably higher than those in the high-level GLA group. Overall, serum GLA levels were strikingly correlated with erythema severity and anxiety/depression.

GLA is a bioactive PUFA biosynthesized through the desaturation of linoleic acid in the liver by the Δ6-desaturase enzyme.^11^ Previous studies revealed that altered levels of GLA would enhance risk of several human diseases, such as diabetes, metabolic disarrangements, and neuropsychiatric disorders.^12^ In addition to its function in lipid metabolism, GLA also poses an anti-inflammatory effect, the ability to repair the skin barrier, and the anti-cancer effect. It was previously reported that patients with atopic dermatitis have lower levels of GLA.^11^ Importantly, A previous study revealed that a supplement of GLA can improve clinical outcomes of rosacea, implicating the possible role of GLA in rosacea.^10^

Rosacea is a highly prevalent skin disease among adults, particularly in younger women.^3^ Despite the advances in the field of rosacea over the past decades, the mechanisms of rosacea are still poorly understood. To our knowledge, few studies focus on the relationship between the changes in serum GLA levels and rosacea. The present results for the first time found that rosacea patients had lower levels of serum GLA. The most common subtype of rosacea is ETR, which remarkably features the erythema that is assessed by PSA and CEA scales.^13^ Subgroup analysis revealed that patients with higher levels of GLA had lower PSA and CEA scores than those with lower-level GLA. Also, it is worth mentioning that the authors found serum GLA levels were negatively associated with erythema, the most common symptom of rosacea. In parallel with the present results, a randomized clinical trial found the supplement of GLA can improve the clinical outcomes of rosacea.^10^ Moreover, the current treatment for rosacea, topical brimonidine, obtains poor efficacy.^14^ To explore whether serum GLA level could influence the efficacy of topical brimonidine, the authors also investigate the predictive value of serum GLA levels for the prognosis of rosacea using a linear regression model. Mechanically, the regression analysis identified that GLA level is a predictive factor for the prognosis of rosacea, with higher GLA levels accompanied by better clinical outcomes. Taken together, the present study and others provided evidence that serum GLA levels are associated with the erythema status in rosacea patients. The mechanism underlying the association might be multifaceted. Besides regulating lipid metabolism, GLA might participate in the pathogenesis of rosacea through anti-inflammation and restoring the skin barrier, which has been validated by previous studies.^10^, ^15^ In systemic inflammation, GLA has been suggested to suppress the upregulation of prostaglandin E2 which plays a crucial role in advancing inflammatory response,^16^ highlighting the potentially negative role of GLA in systemic inflammation.

Rosacea is also suggested to be associated with psychological disorders, especially anxiety and depression.^17^ Indeed, the previous study also demonstrated that rosacea is influenced by anxiety/depression status, and the intervention of anxiety/depression status can strengthen the benefits of drug treatment.^3^ As mentioned above, GLA is associated with the development of rosacea, whereas it remains unclear whether GLA has an association with psychological status. In the present study, the authors utilized GAD-7 and PHQ-9 scales to evaluate anxiety and depression status, respectively. Strikingly, similar to disease severity, the low-level GLA group had worsened anxiety/depression status, which was also correlated with GLA levels. However, further study is required to decipher the mechanism underlying the correlations.

The development of rosacea is attributed to multiple factors, including abnormal immune response, disrupted lipid metabolism, and impaired skin barrier.^18^, ^19^ On the basis of the multifaced factors, several studies, including our own, found limited improvements in rosacea patients.^3^ Therefore, targeting major shared signaling pathways may prove more effective than targeting individual receptors in isolation. As GLA is involved in multiple shared pathways, it might hold promise for treating rosacea.

## Conclusion

In summary, the present study indicates that lower levels of serum GLA in rosacea patients are negatively associated with erythema severity and anxiety/depression status.

## Availability of data and materials

All data generated or analyzed during this study are included in this article and its supplementary material files. Further inquiries can be directed to the corresponding author.

## Financial support

This study was supported by grants from the Chongqing Science and Health Joint Medical Research Project (Reference number: 2023MSXM015).

## Authors’ contributions

Jin-Yi Tang: Contributed to the critical literature review and study concept and design; Wrote the manuscript; Effective participation in research orientation and did the statistical analysis.

Mei-Ling Chen: Contributed to the critical literature review and effective participation in research orientation; Data collection, analysis and interpretation.

Mei Wan: Management of studied cases; Data collection.

Jin-Yu Wei: The propaedeutic and therapeutic conduct of the studied cases.

Tian Qian: Made a manuscript critical review of the literature.

Yu-Kun Fan: The propaedeutic and therapeutic conduct of the studied cases.

Zhi Yang: The propaedeutic and therapeutic conduct of the studied cases.

Jian Fu: The propaedeutic and therapeutic conduct of the studied cases; Effective participation in research orientation.

Jian Li: Contributed to the critical literature review and study concept and design; Wrote the manuscript; Effective participation in research orientation and finally approved the final version of the manuscript.

## Conflicts of interest

None declared.
