# Supplementary material for: Associations of serum gamma-linolenic acid levels with erythema severity and anxiety/depression status in patients with rosacea
Source: An Bras Dermatol. 2023 Dec 6;99(2):189–95. doi: 10.1016/j.abd.2023.01.008 (PMC10943310; doi:10.1016/j.abd.2023.01.008)
Supplement: Supplementary file 1 [file mmc1.docx]

**ABD-D-23-00578 - Supplementary Material**

**Associations of serum gamma-linolenic acid levels with erythema severity and anxiety/depression status in patients with rosacea**

**Subjects and methods**

***Subjects***

A total of 62 rosacea patients and 14 age-matched Normal Controls (NCs) were consecutively recruited from the southwest hospital, the first affiliated hospital to the third military medical university. All rosacea patients were ETR subtype. Rosacea patients were included if they met the following criteria: (1) Reaching the latest diagnostic guide of rosacea; (2) At least 18-years old; (3) Absence of serious infections, cardiac diseases, liver dysfunction and renal disorders. In contrast, patients were not eligible if they met one of the following exclusion criteria: (1) Patients who were allergic to brimonidine; (2) Patients with severe cardiovascular diseases, orthostatic hypotension, cerebral or coronary insufficiency; (3) Patients with severe systemic diseases (myocardial infarction, cardiac failure, cerebral infarction, liver cancer, liver cirrhosis, renal syndrome, acute/chronic renal failure) and hematopoietic disease; (4) Patients with acne, ectopic dermatitis and other facial skin diseases; (5) Pregnant or nursing women. The authors also collected the clinical data of all participants, including age, sex, hypertension, diabetes, etc. In addition, the authors obtained the informed consent form. Rosacea patients received topical brimonidine as the conventional treatment. The present study abided by the principles of the Declaration of Helsinki and was approved by the Ethics Committee of southwest hospital.

***Clinical assessment***

Patient's Self-Assessment (PSA) scale and Clinician Erythema Assessment (CEA) were performed to evaluate the degree of erythema. The details of CEA and PSA scales were presented in supplementary materials (Table S1). Both scales were conducted by a professional dermatologist according to 5-point scales, with higher scores standing for greater severity.

***Psychological evaluation***

The 7-item Generalized Anxiety Disorder (GAD-7) and the 9-item Patient Health Questionnaire (PHQ-9) were conducted to assess the severity of anxiety and depression, respectively. The PHQ-9 contains nine criteria to evaluate degree of depressive disorders. Each of the nine items can be scored from 0 to 3, corresponding to not at all and nearly every day, respectively, and the total scale score ranges from 0‒27. The GAD-7 is comprised of seven items designed to assess generalized anxiety. Analogous to the PHQ-9, each item is marked from 0 (not at all) to 3 (nearly every day). The total GAD-7 scale score ranges from 0‒21.

***Blood sampling and GLA measurement***

Fasting blood was sampled between 07:00 and 09:00 am to follow the cardiac biorhythm at the first visit. After that, blood samples were quickly centrifugated at 3000 rpm, and serum was separated and kept in in -80°C for further analysis. Lipids were extracted from 0.5 mL of serum using methanol and chloroform (1/1 vol/vol). The total lipids, together with pentadecanoic acid (40 μmoL/L) added as an internal standard, were trans-methylated using methanol/3N hydrochloride. Then, GLA levels were determined by gas chromatography-mass.

***Statistical analysis***

All data were analyzed by SPSS 22.0 and graphed by Prism Graphed 9.0. Continuous data were recorded as mean ± SD, while categorical data were presented by number and percentage. To compare the difference between two groups, independent *t*-test was utilized when the data comply with the normal distribution. The Chi-Square test was used to analyze the difference in categorical data. The linear regression model including all 62 rosacea patients was performed to investigate the predictive value of GLA levels for disease severity. Spearman correlation analysis was conducted to analyze the relationship between GLA levels and psychological status. The ANCOVA analysis (longitudinal) was conducted to assess the CEA, PSA, GAD-7 and PHQ-9 scores after 1 month follow-up. A p-value <0.05 was considered statistically significant.

**Table S1** Clinician’s Erythema Assessment (CEA) and Patient’s Self-Assessment.

| **Scores** | **CEA** | **PSA** |
| --- | --- | --- |
| 0 | Clear skin with no signs of erythema | Clear of unwanted redess |
| 1 | Light redness; almost clear, | Nearly clear of unwanted redess |
| 2 | Definite redness; mild erythema | Somewhat more redness than I prefer |
| 3 | Marked redness; moderate erythema | More redness than I prefer |
| 4 | Fiery redness; severe erythema | Completely unacceptable redness |
